# Retrospective study of factors associated with late detection of oral cancer in alberta: A qualitative study

**DOI:** 10.1371/journal.pone.0266558

**Published:** 2022-04-26

**Authors:** Parvaneh Badri, Vickie Baracos, Seema Ganatra, Hollis Lai, Firoozeh Samim, Maryam Amin

**Affiliations:** 1 School of Dentistry, Faculty of Medicine and Dentistry, University of Alberta, Edmonton, Alberta, Canada; 2 Department of Oncology, Cross Cancer Institute, Faculty of Medicine and Dentistry, University of Alberta, Edmonton, Alberta, Canada; 3 Division of Oral Medicine, Pathology and Radiology, School of Dentistry University of Alberta, Edmonton, Alberta, Canada; 4 Oral Medicine/Oral pathologist Division, McGill University Faculty of Dentistry, Montreal, Quebec, Canada; Ruđer Bošković Institute, CROATIA

## Abstract

Oral cancer continues to be diagnosed in advanced stages, giving patients lower chances of survival. The objective of this study was to explore reasons for delayed diagnosis of oral cancer in Alberta. A retrospective qualitative design was implemented through seven steps suggested for conducting a narrative clinical document. Data was retrieved from the Alberta Cancer Registry database between 2005 and 2017. A sample of initial consultation notes (ICN) of oral and oropharyngeal cancer patients were identified through a purposeful sampling method and added to the study until saturation was achieved. A deductive analysis approach inspired by the model pathways to treatment health care provider (HCP) was employed. From the 34 ICN included in our analysis, five main categories were identified: appraisal interval, help-seeking interval, diagnosis interval, pre-treatment interval, and other contributing factors such as health-related behaviours, system delay, and tumor characteristics. These factors negatively contributed to early detection of oral and oropharyngeal cancers and affect treatment wait time with patients, providers, and the healthcare system. Patient’s lack of awareness, provider’s oversight and prolonged access to care were the main reasons of delay in cancer diagnosis and management in our study. A sustainable plan for public awareness interventions and implementation of a solid curriculum for medical and dental students is needed to enhance their related knowledge, competence in clinical judgement, and treatment managements.

## Introduction

Being the 11th most common cancer worldwide, oral cancer is a major public health concern [1, 2]. The issue of delayed detection of oral cancer is gaining increased attention by clinicians who believe that detecting oral cancer at an early stage is the most effective means of reducing rates of the disease morbidity [3]. For decades, the late detection trend for oral cancer has remained a challenge for health professionals and authorities, as it is associated with a relatively poor prognosis (a five-year survival rate of 50%-60%) and lower quality of life [4]. Late detection of oral cancer also leads to higher therapy costs for survivors [5].

To date, multiple factors have been investigated in the literature as independent prognostic markers for oral cancer such as age, co-morbidity, immunological or nutritional status, size/location of the tumor, nodal status, oncogene expression, proliferation markers, and tumor DNA content [6, 7]. In addition, numerous modalities have been used to detect precancerous lesions. These include periodic conventional oral cavity examination for symptomatic and/or non-symptomatic non-healing oral mucosa lesions, oral cytology, optical technologies, fluorescence imaging and more [8]. Other factors extracted from patients’ histories and activities, include smoking, recreational drug use and alcohol consumption, as well as genetic predisposition and past/present oral HPV infection, immunodeficiency, and poor oral hygiene [9, 10].

Selective opportunistic screening has been introduced in some studies as a more realistic and effective solution versus routine screening, particularly in the detection of oral squamous cell carcinomas in a non-symptom-driven examination [11, 12]. This approach has led to diagnosis at an earlier stage, similar to the significant early detection of oral cancer in patients who attend regular dental visits [13, 14]. In their study, Seoane-Romero and colleagues illustrated that delay in diagnosis is not necessarily associated with advanced stage at diagnosis, nor is obtaining a fast diagnosis a guarantee of an early-stage tumor [7]. Nonetheless, any delay in cancer diagnosis is not generally desirable [7]. According to the study, poor tumor differentiation (e.g., deeming the tumor to be biologically more aggressive) is an independent risk factor for diagnosis at advanced stages [7].

Oral cavity and oropharyngeal cancers (OPC), which have been grouped under the general term oral cancer (OC), have shown epidemiological changes [15]. Older men of low socioeconomic status used to be the main victims of the disease; however, in the past decade, many young people including women, and higher socioeconomic classes are being diagnosed with OPC [15]. This shift has added to the complexity of challenges that patients and healthcare systems have faced for decades because of OC and OPC aetiologies, clinical presentations, management, and survival rate differences.

Among Canada’s ten provinces, Alberta is positioned fourth after Ontario, Quebec and British Columbia for oral cancer incidence and related death prevalence [16]. Our previous studies have shown that 45.2% of OC and 82.4% of OPC cases in Alberta are diagnosed in stage IV, with a 47.9% mortality rate. Therefore, the primary research question of this retrospective study was ‘what were the challenges experienced by clinicians and patients regarding early detection of oral cancer in Alberta?’ The objectives then were to better understand the reasons for delayed diagnosis of oral cancer in Alberta as well as the difficulties experienced by patients and healthcare professionals dealing with oral cancer, using recorded medical Initial Consultation Notes (ICN). A model pathway for cancer treatment was used to illustrate the key determinants to cancer outcome. Using a theoretical model could help to mitigate diagnosis delay.

## Materials and methods

A retrospective qualitative design was implemented using seven steps suggested for conducting a narrative clinical document analysis [17–19]: **1.** Identifying the research question; **2.** identifying the appropriate data source; **3.** devising a data extraction plan; **4.** extracting the data; **5.** checking for errors; **6.** analyzing the data; and **7.** archiving and disseminating the findings. Ethics approval was obtained from the Health Research Ethics Board of the Alberta Cancer Committee (Ethics ID# HREBA.CC-17-0370).

Access to personal identifiable health information was requested through ethics application. Upon review, the HREBA–Cancer Committee waived consent as it was demonstrated to be impractical, unreasonable and not feasible to obtain. To ensure the anonymization of samples and protect their confidentiality, we made every effort to remove all identifiers.

A purposeful sample [17, 20, 21] of medical charts constituting the very first Initial Consultation Notes from the multiple consultation list of each case of OC and OPC patients listed in the Alberta Cancer Registry (ACR) between 2005 and 2017 was included in this study. The Alberta Cancer Registry data set mainly includes data from oncology departments. The initial consultation data from family physicians, dentists, and other specialties are scattered throughout numerous accessible and inaccessible resources. The consultation notes were made by oncology department clinicians. We selected the very first visits and history-taking notes.

The following tumor location/site were categorized according to the topographical codes in the International Classification of Diseases for Oncology, 3rd edition in the study, ICD-0 3. OC sites included lip (C00.3-C00.9), oral tongue (C2.0-C2.3, C2.8 and C2.9), gum (C3.0-C3.0), floor of mouth (C4.0-C4.9), palate (C5.0-C5.9) and other and unspecified parts of the mouth (C6.0-C6.9). OPC sites included base of tongue (C01), lingual tonsil (C2.4), tonsil (C9.0-C9.9), oropharynx (C10.0-C10.9), pharynx not otherwise specified (C14.0) and Waldeyer ring (C14.2). External upper and lower lip (C00.0-C00.1), parotid gland (C07.9) and other and unspecified major salivary gland tumors (C08.0-C08.9) were excluded.

In contrast to probability sampling in a quantitative study, in qualitative inquiries, purposeful sampling is the most appropriate technique for recruiting participants for interviews or selecting medical charts for a qualitative analysis [17, 20, 21]. For our sampling, the charts were selected based on the maximum variation of nonprobability sampling strategy [20]. This strategy assists in identifying essential features and variable aspects of the study phenomena among varied contexts [22]. For the purpose of the present investigation, a range of Initial Consultation Notes of OC and OPC patients was selected according to age, sex, pathological oral site, geographic zone, annual income, clinical stage, and vital status [23]. There is a growing consensus in the literature for defining stages I and II as early stage and stages III and IV as late stage of oral squamous cell carcinoma with poorer prognosis and survival rates [7, 23]. In this study, we only included stage IV patients since there were higher numbers of cases diagnosed at stage IV (OC: 42.2%; OPC: 82.4%) compared to those recorded at stage III (OC: 9.9%; OPC: 10.0%) [24].

Assessment of the data source in terms of accuracy and completion was implemented. The Initial Consultation Notes (ICN) retrieved from the medical charts of patients were reviewed, coded, and crosschecked independently by two reviewers (PB and FS) and they reached to consensus over disagreements through discussions. Subsequently, 10% of total reviewed sample verified by a third reviewer using predefined criteria to determine whether they were accurate and/or complete [25].

Data collection was considered complete at data saturation when no new data emerged to answer the study’s research questions [17]. Descriptive analyses were performed to assess the inaccuracies and incompleteness of each chart information field using a comparison method of data [25].

A series of open-ended questions were employed (**Table 1)** to assist in extracting data and coding the collected information based on the objectives. These questions clearly indicated that the data elements needed to be extracted from the ICN of the patients. Extraction of data was conducted in accordance with the instrument devised and the element definitions agreed upon by the research team.

**Table 1:** Data extraction guide.

	Open-Ended Questions
1	What brought the patient for the first assessment (first symptoms, if any) and when?
2	What particular underlying risk factors were detected?
3	Did the clinician identify the symptom as potentially malignant or pre-malignant?
4	What tests were ordered?
5	What was the clinician’s first attempted intervention?
6	What was/were the outcomes of the attempted intervention(s)?
7	What was the time from onset of the first symptom to contacting a health care professional of any kind?
8	What was the time from first contact with a health care professional to the date of definitive diagnosis?
9	What were the barriers/challenges causing delay in diagnosing the cancerous lesion?
	a) Experienced by whom?
	b) Associated factors at any level?

Two raters coded the data and evaluation of inter-rater reliability was performed. The various terminologies used were defined for clarification. A small sub-sample (approximately 10% of the total) was reassessed to check agreement with the previously coded data and to detect any inaccuracies. The data were analyzed qualitatively using deductive manifest content analysis [17], which was accompanied by a descriptive statistical analysis of demographic characteristics of patients whose charts were included in the study. Deductive content analysis is an analytical method that aims to investigate a new similar context using existing categories, theories, models, and concepts [26]. In contrast to latent content analysis, which refers to the interpretation or underlying meaning of content or interview, manifest content refers to evidence directly seen, such as words in a document requiring the least amount of interpretation [26].

**Fig 1:**
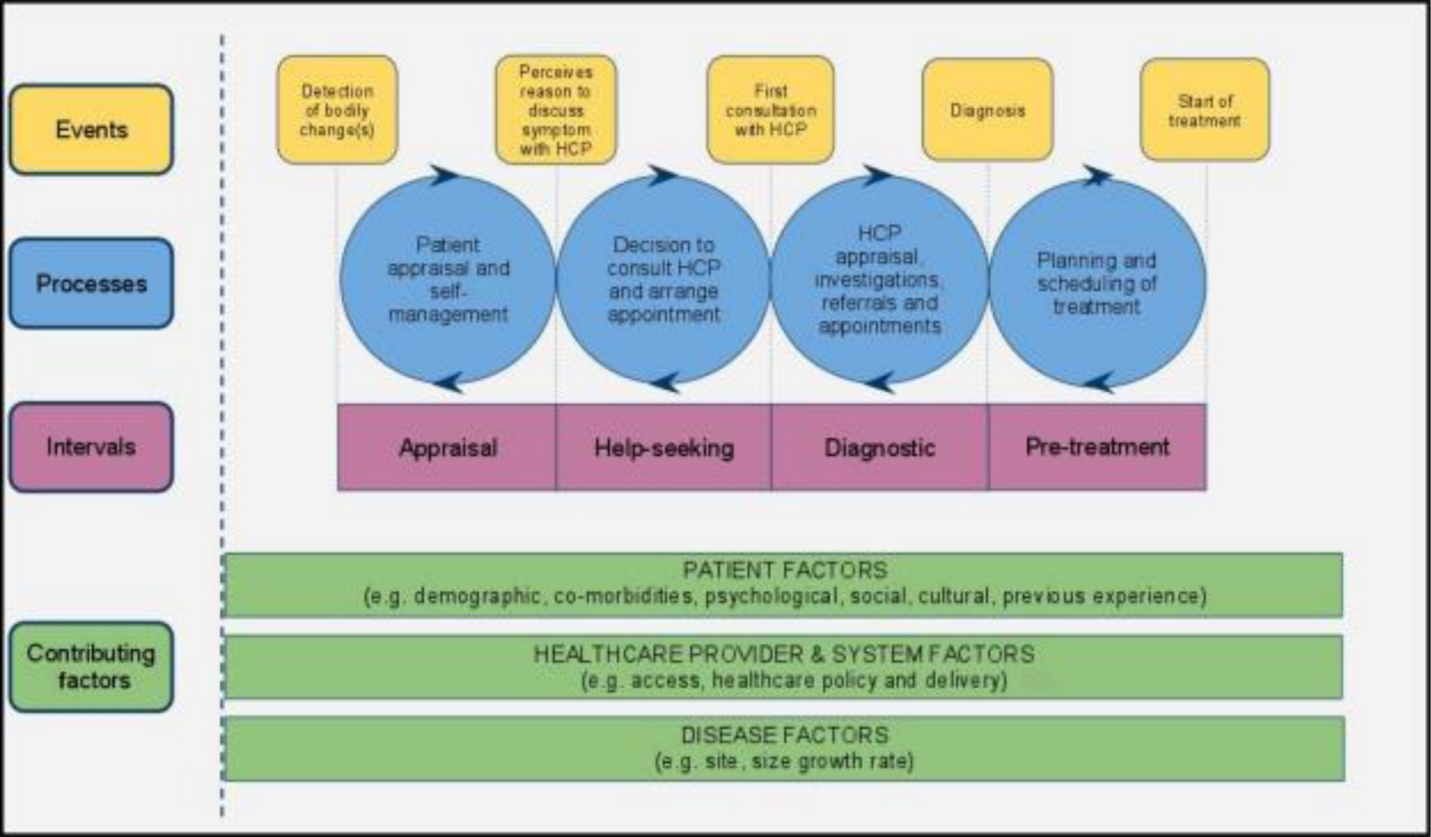
Model of pathways to treatment health care provider [27].

The Model of Pathways to Treatment HCP for cancer was used to analyze and communicate the unknown context experienced by oral cancer patients generated from the ICN documents [27]. This model is one of the most cited theoretical models for cancer diagnosis modified from the original 3-stage Safer et al model (1979), which comprised ‘appraisal delay’, ‘illness delay’, ‘utilization delay’ [27]. In the original Andersen model, the ‘utilization delay’ was expanded to ‘behavioural delay’, ‘scheduling delays, and ‘treatment delay’[27]. The model has been applied in different cancer studies, including oral cancer [28, 29]. However, the use of the term “delay” was found to be inappropriate because the role of factors associated to the lesion (i.e., the site and aggressiveness of the tumors) or healthcare system might be ignored [29]. Therefore, a new modified model was proposed that included four intervals: *appraisal interval* (the period when a patient first recognizes his/her symptoms and perceives that a HCP should be consulted); *help-seeking* (the period when the patient first deems it necessary to seek help and makes the first consultation appointment with an HCP); *diagnostic* (the period from the first HCP consultation to diagnosis); and *pre-treatment* (the period from confirmation of diagnosis to initiation of treatment) **(Fig 1).**

The deductive manifest content analysis was performed in three phases: preparation, organizing, and reporting [30, 31]. In the preparation phase, the ICN identified as the unit of analysis were analyzed line by line, excluding the detailed description of the treatment, which was outside of the scope of this study. Two authors (PB and FS) read the ICN several times in order to become familiar with the provided information, make sense of the data, and learn “what is going on” [32]. The repeated review of the content also helped the two coders to highlight the key meaningful units based on our research questions.

**Fig 2:**
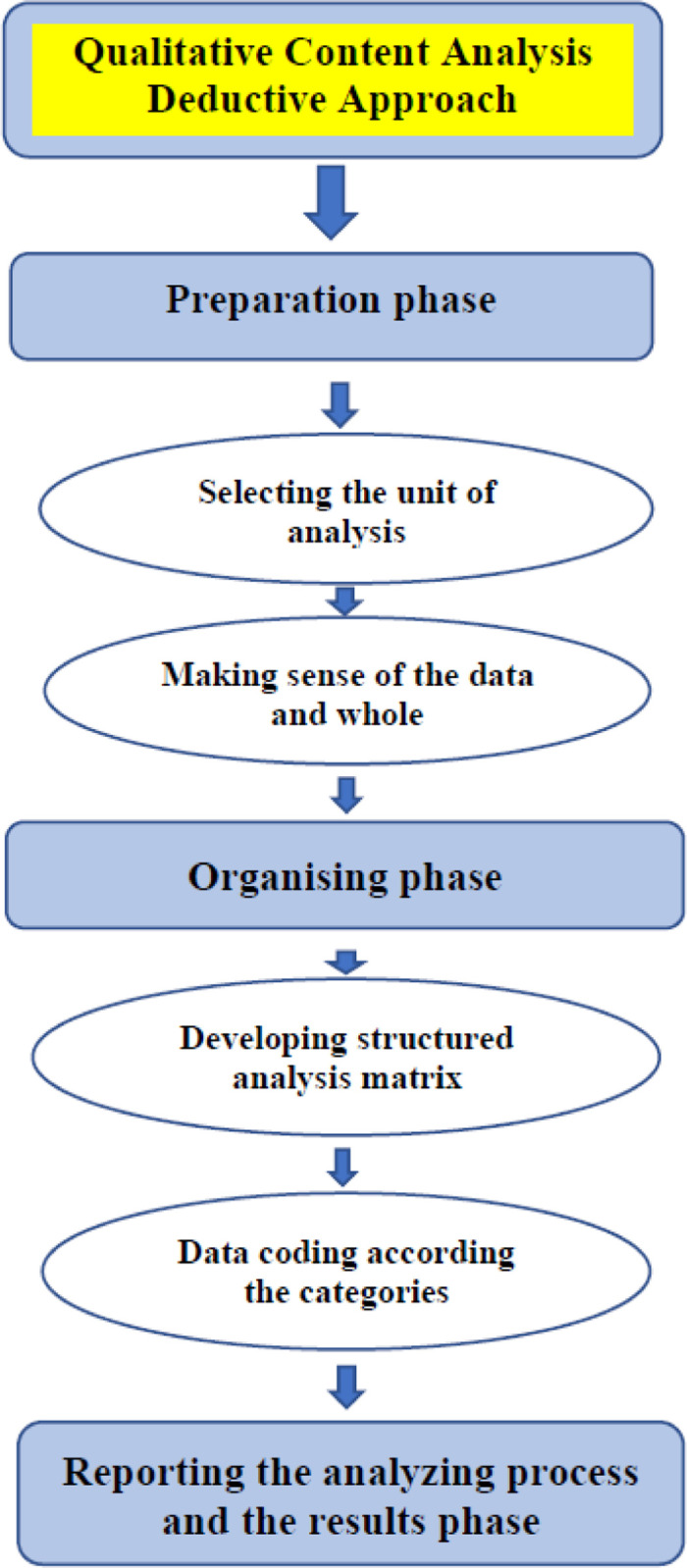
Phases of preparation, organizing, and reporting in the deductive content analysis process [31].

In the organizing phase, a structured categorization matrix was built to reflect the research questions through the model of pathways to treatment HCP. The two coders then reached an intercoder agreement for generating the data coding; the agreement involved organizing the coding under the defined categories in order to describe multiple angles of phenomena that are of interest to this study [20, 33]. In the reporting phase, the five identified categories enhanced knowledge in order to better understand the challenges experienced by clinicians and patients regarding early detection of oral cancer in Alberta [34] **(Fig 2).**

## Results

Of the total 1,987 oral cancer patients registered at stage IV at the Alberta Cancer Registry between 2005 and 2017, 34 Initial Consultation Notes were retrieved and included in our analysis. We met data saturation at the 30^th^ Initial Consultation Note, but the data collection continued for four more documents to ensure there were no additional new data relevant to the study phenomena. The patients’ mean (SD) age was 56.3 (14.31) and ranged from 32–90 years; 70.5% were male and 85.2% were living in urban areas. The range of household income was between 27,336 and 138,161 CAD. At the time of data collection, 26 of the 34 included patients (76.4%) were deceased **(Table 2).**

**Table 2:** Descriptive analysis.

Case No/Pt. ID	Age (Year)	Sex	Anatomical cancer sites	Rural/Urban	Median household income $ CAD	Cancer Stage AJCC6/7	Number of months from assumed first symptom (s) to first clinician visit	Number of visited clinicians prior to oncologist	First Health Care Provider
Case 2	30–49	M	Floor of mouth	Urban	38876	IVA	<1	3	Physician
Case 5		F	Floor of mouth	Urban	60127	IVA	48	Unknown	Unknown
Case 6		M	Base of tongue	Urban	55403	IVC	<1	Unknown	Most likely Physician
Case 14		M	Palate	Urban	68382	IVA	0	Multiple from 2003 to 2010	Periodontist
Case 15		M	Palate	Urban	137218	IVA	<1	Unknown- At least 2	Unknown
Case 20		M	Tongue, other & unspecified	Urban	48051	IVNOS	8	2	Physician
Case 21		F	Tongue, other & unspecified	Urban	31002	IVB	<1	2 or more	Physician
Case 26		M	Mouth, others & unspecified	Urban	149323	IVA	2.5	2	Unknown
Case 27		M	Mouth, others & unspecified	Urban	41366	IVA	0	2?	Dentist
Case 28		M	Mouth, others & unspecified	Urban	117595	IVA	4	Unknown	Unknown
Case 30		M	Base of tongue	Urban	75959	IVNOS	5	3?	Unknown
Case 32		M	Base of tongue	Urban	29449	IVC	6	2? (Inpatient)	Physician
Case 34		F	Base of tongue	Urban	47342	IVA	14	3	Physician
Case 1	50–69	F	Floor of mouth	Urban	55468	IVA	1	3	Dentist
Case 3		M	Floor of mouth	Rural	47257	IVC	<1	2	Dentist
Case 4		M	Floor of mouth	Urban	27336	IVA	<1	2	Unknown
Case 8		F	Base of tongue	Urban	97429	IVB	2	3	Dentist
Case 9		M	Gum (Gingiva)	Urban	36491	IVNOS	<1	2	Physician
Case 16		M	Palate	Urban	27450	IVB	3	Unknown	Most likely Physician
Case17		F	Gum (Gingiva)	Urban	54666	IVNOS	<1	2	Most likely Physician
Case18		F	Gum (Gingiva)	Urban	74563	IVA	<1	3	Dentist
Case 19		F	Tongue, other & unspecified	Urban	36403	IVC	1	2	Dentist
Case 22		M	Mouth, others & unspecified	Urban	49161	IVNOS	<1	4	Physician
Case 23		M	Mouth, others & unspecified	Rural	47351	IVC	3	Unknown (2?)	Unknown
Case 24		M	Mouth, others & unspecified	Rural	29870	IVB	<1	Unknown	General Surgeon
Case 29		M	Mouth, others & unspecified	Urban	40844	IVA	3	2	Physician
Case 33		M	Base of tongue	Urban	110266	IVB	1	3	Physician
Case 7	70–90	M	Base of tongue	Urban	103738	IVC	1	2	Physician
Case 10		F	Gum (Gingiva)	Urban	114658	IVC	1	2	Physician
Case 11		M	Palate	Urban	29283	IVC	0	1	Physician In-patient
Case12		F	Palate	Rural	49588	IVB	240	Unknown	Unknown
Case 13		M	Palate	Rural	47024	IVA	22	Multiple, at least 3	Physician
Case 25		M	Mouth, others & unspecified	Urban	138161	IVB	3	Unknown	Most likely Physician
Case 31		M	Base of tongue	Urban	103738	IVC	3	2	Physician

**Fig 3:**
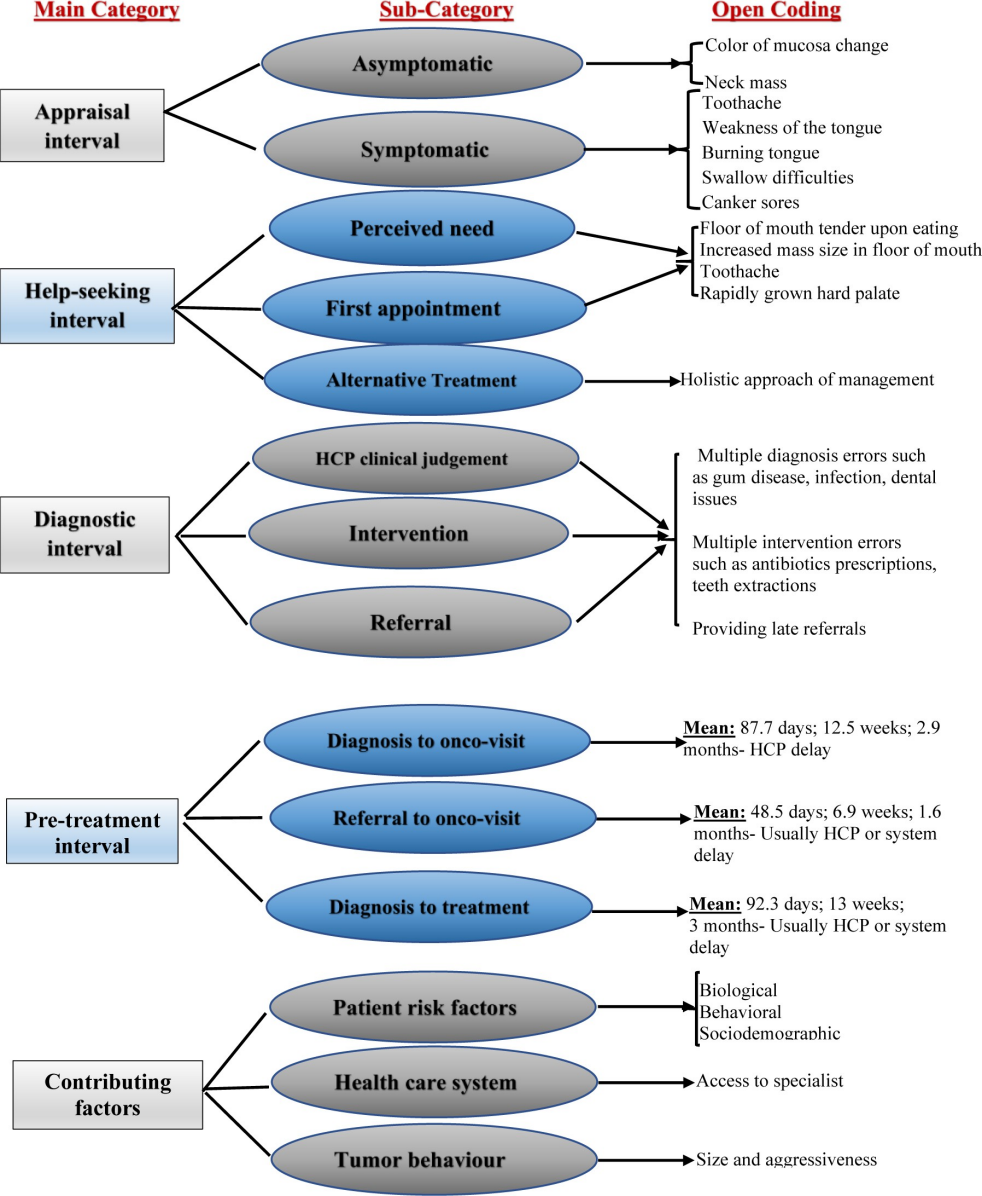
Deductive coding the data to the categorization matrix.

Using the model of pathways to treatment HCP, the retrieved information was grouped into five categories: ‘patient appraisal interval’, ‘help-seeking interval’, ‘diagnostic interval’, ‘pre-treatment interval’, and ‘contributing factors” **(Fig 3).**

### Appraisal interval

In this interval, diagnosed patients noticed the various types of asymptomatic and symptomatic changes for the first time in their head and neck area. Some of the asymptomatic presentations included abnormal growths in different anatomic areas such as floor of the mouth, gingiva, submandibular, cheek (buccal mucosa), and/or white changes of the mouth, while symptomatic presentations included toothaches, sore throat, ill-fitting dentures, canker sores, burning sensation, and difficulty swallowing. For instance, a 49-year-old male patient noticed a painless lesion on his floor of the mouth for a year without seeking medical attention. Similarly, a 47-year-old male patient, while aware of an enlarging neck mass for a period of time, did not perceive the need to consult a healthcare provider immediately. Patient’s lack of awareness repeatedly caused delays in care-seeking until the lesion became symptomatic:

*“…*. *she started to notice weakness in her tongue and difficulty swelling as well as voice changes…”* [Initial Consultation Notes, Case 8]

*“Mr*. *… noticed a burning sensation on his tongue*, *he thought this might have been due to his inhalers for COPD…*..*”* [Initial Consultation Notes, Case 20]

*“He… first noticed a canker on the left buccal mucosa*,,*…*.., *he initially attributed this canker to[his] gutka or bitel nut) [used for long time]…”* [Initial Consultation Notes, Case 26]

For most cases, there was a gap period from the time the patient noticed the changes to the time they recognized the change as a health issue requiring a medical consultation **(Table 3).**

**Table 3:** Main categories information.

Case No/Pt. ID	Days: Accumulated appraisal and help-seeking intervals *Average*: *350 Median*: *31*	Days: Diagnostic interval *Average*: *184 Median*: *38*	Risk factors:				Specialist access A = 56% B = 44.1% C = 20.5% O = 23.5%
			Family history of cancer	Heavy smoker and alcohol drinker	History of radiation	Depression	
Case 1	28	210					A
Case 2	14	348	✔	✔			A
Case 3	14	30		✔		✔	O
Case 4	14	13	✔	✔			A & C
Case 5	1415	17		✔			A & B
Case 6	14	247		✔			A & B
Case 7	36	87	✔	✔			B
Case 8	64	168		✔		✔	O
Case 9	14	18		✔	✔		A & B
Case 10	30	78					A & C
Case 11	0	77	✔		✔	✔	C
Case 12	7215	99		✔			O
Case 13	1065	1083		✔		✔	A
Case 14	0	2722	✔				A & B
Case 15	14	213					A & B
Case 16	91	19		✔			A & C
Case 17	14	21		✔		✔	A & C
Case 18	14	15					B
Case 19	31	26		✔			A
Case 20	275	93		✔			O
Case 21	14	128	✔	✔			A & C
Case 22	14	96	✔	✔			B
Case 23	90	6		✔			A & B
Case 24	14	8	✔	✔			A & B
Case 25	89	139		✔			O
Case 26	106	20		✔			O
Case 27	0	35					A & B
Case 28	151	19		✔			A
Case 29	92	8	✔	✔			B & C
Case 30	182	91	✔	✔			A & B
Case 31	92	31	✔	✔			B
Case 32	183	29	✔	✔			B
Case 33	31	40		✔			O
Case 34	485	29	✔				O

A: Specialist A; B: Specialist B; C: Specialist C; O: Others.

### Help-seeking interval

The patients included in this study came from various sociodemographic backgrounds and demonstrated a broad range of health behaviours and concerns. As a result, they responded differently when they noticed unusual asymptomatic or symptomatic changes in their head and neck area. While some patients sought help immediately, for others, it took them from 14 to 1,000 days to seek help. For some patients, it even took much longer (7,215 days) to *perceive* a reason to *schedule an appointment* and discuss changes with an HCP or to seek *alternative help*, such as a holistic approach. For instance, a lady working evenings at a large store noticed a painless growth on her floor of the mouth. She waited for about four years (1415 days) until she decided to see an HCP. In addition, we found that family physicians were the first HCP seen in almost 50% of patients compared to 20% who saw a dentist **(Table 2).**

*“Mr*. *…*. *began having a toothache about two months ago [Oct*. *…]*. *He was seen by a dentist [Oct*. *…] and at that time an intraoral lesion was seen…(14 days)”* [Initial Consultation Notes, Case 3].

*“Her history dates back to June when she started to notice weakness in her tongue and difficulty swallowing as well as voice changes*. *She originally saw a dentist in August … (64 days)” …”* [Initial Consultation Notes, Case 8].

*“Ms*. *…*.*who noticed some discomfort and fullness in the left submental area …*. *This progressed to some earache on the left side and some sensation of fullness in the left ear*. *She subsequently had medical consultation [after 4 months]”*.*… (485 days)”* [Initial Consultation Notes, Case 34].

*“…*, *who is a pleasant lady*, *[over 50 years old] who has had a left mandibular gingival mass for over 2 years …originally biopsied as verrucous carcinoma*. *At that time*, *she was offered a surgical intervention*, *but based on her personal beliefs and based on the recommendation from her holistic sources*, *she opted holistic approach to this mass for which she thought was an infection*. *Over the past 2 years the mass has slowly grown and has grown more progressively and worse over the past 2 months… cause her significant trismus …and ulceration of the skin …overlying her left mandible*. *This causing her to get significantly worse and had her start doubting her holistic approach to her mass…*..*)”* [Initial Consultation Notes, Case 18].

In our study, the median time from when a patient noticed the first symptom(s) to the first scheduled appointment was 31 days. However, the actual *help-seeking interval* was from the time the patient perceived the need for care to the first consultation. Therefore, it was the perceived need that led to the patient’s first medical visit with an HCP **(Table 3).**

### Diagnostic interval

The competence of the first HCP (dentists, family physicians) who performed the initial screening and detected the potentially malignant lesions in the oral cavity as well as timely referrals were essential contributing factors to definitive diagnosis and treatment outcomes.

*“Mrs*. *…had some* *dental difficulties for the past one year* *(about January)*. *A far as she was aware*, *this was due to some abnormalities within the gum*, *which been attributed to previous* ***antibiotic therapy*** *and* *extraction of her teeth* *on the lower right side in March or April …*, *and since that time she had been* *experiencing ongoing pain*, *and not healing*. *In September*, *she was* *referred to an oral surgeon* *because she had developed some swelling along the right mandible and appeared to have an infection at the site of her previous surgery [Extraction site]*. *Therefore*, *a debridement was performed*. *Pathology from debridement identified a well-differentiated squamous cell carcinoma in September …*. *However*, *according to Mrs*. *…* *the pathology result was not received until November …**” (Diagnosis interval*:*210 days)*- [Initial Consultation Notes, Case 1].

*“Mr*. *… over 30 years old’s history began about* *one year ago* *when he noticed a* *burning sensation on his tongue*. *He thought this might have been due to his inhalers for COPD*. *However*, *he* *began to notice a lump in the right side of his tongue about 8 to 9 months ago* *and this has slowly grown in size*. *Four to* *five months ago*, *he developed lumps on the right side of his neck and under his mandible*. *By this point*, *he was also developing some* *otalgia*, *and was having difficulty with swallowing* *and* *with speech* *due to the size of the mass*. *He went to see his family doctor [about one month later]* *and was* *treated with* ***antibiotics***, *but this did not have any effect…*. *As there was* *no improvement*, *the patient was subsequently* *referred to* *Dr*. *…*… *a biopsy was completed [four months later…*, *confirming a moderately differentiated squamous cell carcinoma*. *(Diagnosis interval*:*93 days)*—[Initial Consultation Notes, Case 20].

“Mr. … [younger than 50],…..who noticed a lump in his left neck last October. This was painless. This was gradually growing in size. He denies any changes in voice, swallowing …. His biopsy done in …., …, by Dr. …. back with no evidence of dysplasia or malignancy. I will request Dr…. to take this patient back to the OR and do some deeper biopsies to rule out or confirm malignancy at the base of tongue on the left side.

*[Three months later] Squamous cell carcinoma arising from the left tongue base*. *Diagnosis made on excisional biopsy from an ipsilateral neck node seen on PET/Ct*. *Biopsy from the neck revealed p16 positive disease*.*” (Diagnosis interval*: *91days)* —[Initial Consultation Notes, Case 30].

Lack of knowledge of early signs and symptoms of head and neck cancer, misdiagnosis of the condition resulting in inappropriate managements, and late referrals led to unnecessary long intervals for confirmation of the final diagnosis of cancer. Improper choice of interventions and medical tests also resulted in late diagnosis. In contrast, competent professionals with accurate diagnostic and management abilities could make the diagnostic confirmation interval shorter **(Table 3).**

### Pre-treatment interval

Our study revealed a delayed interval from the definitive diagnosis date to receiving first treatment. To achieve a better understanding of this interval, we looked at three periods including the total number of days from diagnosis date to the first oncologist consultation date; days from receiving a referral to the first oncologist consultation date; and days from diagnosis date to treatment initiation date. Of the 34 cases, in nine cases, the number of days from receiving a referral to the first oncologist consultation date was missing **(Table 4)**.

**Table 4:** Pre-treatment interval related data.

Number of Cases: 34	1	2	3	4	5	6	7	8	9	10	11	12	13	14	15	16	17	18	19	20	21	22	23	24	25	26	27	28	29	30	31	32	33	34
**Days from diagnosis to oncologist visit** *Average*: *88 Median*: *50*	**154**	**30**	**28**	**102**	**56**	**111**	**50**	**520**	**18**	**49**	**7**	**39**	**57**	**45**	**16**	**36**	**119**	**703**	**85**	**15**	**21**	**67**	**63**	**91**	**44**	**36**	**99**	**185**	**15**	***-40***	**50**	**43**	**53**	**15**
**Days from referral to first head and neck consultation** *Average*: *49 Median*: *15*	**NA**	**NA**	**2**	**51**	**NA**	**48**	**15**	**NA**	**3**	**21**	**NA**	**NA**	**17**	**15**	**3**	**NA**	**31**	**14**	**31**	**365**	**126**	**31**	**90**	**14**	**152**	**12**	**4**	**130**	**8**	**10**	**15**	**NA**	**NA**	**6**
**Days from diagnosis to treatment initiation** *Average*: *92 Median*: *56*	**105**	**109**	**34**	**129**	**50**	**96**	**81**	**23**	**-**	**-**	**39**	**27**	**68**	**77**	**91**	**56**	**124**	**769**	**-**	**53**	**54**	**85**	**32**	**-**	**61**	**41**	**159**	**125**	**53**	**2**	**51**	**49**	**-**	**35**

NA: Not available; (-): No treatment; *-40* (the diagnosis confirmed 40 days after first oncologist appointment).

### Contributing factors

In addition to the four identified intervals, three other contributing factors that seemed to influence early detection of oral cancer included those related to the patient, providers and healthcare system, and tumor behaviour.

#### Patient factors (biological, behavioural and sociodemographic)

Our findings captured multiple risk factors reported by patients in this study. This risk factors are categorized as *biological*, such as having a past history or a family history of cancer along with comorbidities; *behavioural*, such as a long history of smoking tobacco/recreational drugs and alcohol consumption. Patients with risk factors are at higher risk for developing cancer and should be a priority for preventive screening to avoid delay; and *sociodemographic*, such as older age, living alone, being divorced/never married, and low socioeconomic status. For example, case 2 was a male with a 37-year history of tobacco and alcohol consumption and his mother died of carcinoma of the stomach. These accumulated factors positioned him at high-risk for developing oral malignancy. Case 11, on the other hand, had a history of divorce and lived alone for 34 years. He also had asbestos exposure and his mother died of lung cancer. A combination of biological and sociodemographic factors increased his risk for cancer.

*“Mr*.*…*. *is currently homeless*, *though he has been in Gunn*, *Alberta for alcohol detoxification*. *He is now an inpatient at the …… under ENT*. *He has a history of alcohol abuse and continues to smoke one to a quarter pack daily*.*”* [Initial Consultation Notes, Case 3]

*“The patient has a past medical history of celiac disease as well as prostate cancer treated with hormone therapy*. *The patient lives in ……in a house with a friend*. *He was smoker quit two years ago*. *Prior to that he had a 50-pack-year smoking history*. *…*. *Patient states that he does drink approximately five drinks per week*.*”* [Initial Consultation Notes, Case 7]

*“The patient [older than 50 years] who lives on his own in an apartment*. *He was previously a …*.. *He has a 40-pack-year history of smoking*. *…*. *he does have a previous history of alcohol abuse*. *Previous history of radiation to the left side of the head*. *…*. *he has previously smoked crack and marijuana*. [Initial Consultation Notes, Case 9]

#### System delay

Timely access to a healthcare provider was found to be important for a better treatment outcome and survival in patients diagnosed with oral cancer. In addition to the already reported increased diagnostic and pre-treatment intervals, our findings identified only two specialists who were in charge of our study cohort, resulting in high patient loads and long waiting periods. **(Table 3).**

#### Tumor behavior

Tumor characteristics such as size, location, invasive behaviour and metastasis are important contributing factors to diagnosis and survival rate. As well, the aggressive behaviour of certain malignancies might cause unwanted outcomes, even for cases that are diagnosed at an early stage and managed in a timely manner.

*“[Her] husband recall that she admitted to the …*. *…*, *presenting with …*.. *At the time it was noted that she had…*.. *At a subsequent dental visit*, *she was advised to seek medical attention for a suspicious tongue lesion*. *[one month later] … she presented to …*. *Hospital*, *and was admitted to the ENT ward…*.*[three days after]*, *a biopsy …performed demonstrating a p16 negative*, *moderately differentiated squamous cell carcinoma*. *Upper endoscopy on the same day demonstrated invading the entire right lateral tongue squamous cell carcinoma*, *extending into the palate*, *base of tongue*, *tonsil*, *right lateral pharynx*, *piriform*, *epiglottis*, *vallecula*, *and root of tongue*, *right lateral pharynx… (Diagnosis interval*:*26 days)* —[Initial Consultation Notes, Case19].

*“Mr*... *[younger than 50 years] followed up by a [oral specialist] for a premalignant lesion of lichen planus of the maxillary gingiva and the premaxillary bone*. *This had been biopsied multiple times [for seven years]*. *The last biopsy … demonstrated squamous cell carcinoma*. *He was otherwise asymptomatic*. *He does complain of some TMJ pain and some pain across the premaxilla and bleeding from his gums*. *Recently*, *periodontal work has revealed losing teeth of his premaxilla and also of the dental implant placed in his anterior teeth*. *(Pre-treatment interval*: *8 days)* —[Initial Consultation Notes, Case 14].

## Discussion

Using the model pathways to treatment HCP, we explored challenges experienced by patients and healthcare providers toward obtaining earlier detection of oral and oropharyngeal cancers in patients in Alberta [27]. Our findings showed a median time of 31 days from the onset of symptoms until the patient perceived a need for a consultation with a healthcare professional (HCP) and the subsequent booking of an appointment. This average is far lengthier than the 31 to 90 days widely reported in the public health literature as the typical threshold, which itself has been criticized for being too long [35–37]. The commonly reported average patient’s delay for OC and OPC from first symptom to first HCP consultation is 105 to 165 days while three months is enough for squamous cell carcinoma to double in size [38]. However, the patients’ Initial Consultation Notes lack an exact indication of the time from when a patient noticed the changes, the time they perceived the need to consult an HCP (*patient appraisal interval*), and when the actual care-seeking occurred (*help-seeking interval*).

Our data demonstrates transitional health-related behaviours by patients consisting of lack of attention at the asymptomatic stage to the symptomatic aspect of malignant changes, which ends at the first consultation. Similarly, Scott and colleagues in their systematic review reported that patient’s delay was mostly due to not seeking care until the lesion became symptomatic [39]. The literature supports the complexity and multifactorial reasons for what causes a longer *appraisal interval*. This includes the lack of symptoms associated with oral malignancies at early stages; the patient’s lack of knowledge about early manifestations of oral cancer; restricted access to HCPs; established health-related behaviour and self-treatment, with or without a pharmacy consultation; socioeconomic factors; and psychological factors such as individuals’ symptom interpretation, disclosure of symptoms to others, and social priorities [38, 40–42].

Diagnosis delay and the interval from a patient’s first consultation visit to the definitive confirmation of the cancer diagnosis (*diagnostic interval*) has been studied by several researchers [36, 43]. In our study, the diagnostic interval range for patients was 6 to 2,722 days (mean 183.5), which is considerably longer than the 14–21 weeks (98–147 days) and 15.4 weeks (107.8 days) reported for diagnostic delay in two other reviews published in 2014 and 2016, respectively [36, 38]. Furthermore, we identified multiple misdiagnosis, inappropriate antibiotic prescriptions, extraction of teeth caused by clinical misjudgments and referral delays that led to several back and forth appointments and subsequent delays in cancer diagnosis. Other diagnostic delays were caused by the health care provider’s recommendation of superficial/incisional biopsy rather than an in-depth/excisional biopsy. While there is considerable evidence in support of our findings for delay caused by professionals [36, 38, 44], Rogers and colleagues, in their study conducted in the United Kingdom, reported that 78% of the cases were referred to specialists on the same day of the patient’s first visit [45].

In our study, 53% of patients approached a family physician for their first consultation as compared to 17.6% who visited a dentist. The high cost for a dental visit could be a reasonable explanation for this choice. According to the literature, family physicians are less familiar with oral lesions, which can result in poorer screening, misdiagnosis, and delayed referrals to specialists, all of which would negatively affect early diagnosis of the lesion [8]. Unlike family physicians, dentists have better training and more knowledge about oral lesions and oral pathology. However, it appeared from our study that oral examinations performed by dentists were not systematic and focused more on teeth or denture-related soft tissue rather than on high-risk anatomical areas such as floor of the mouth and cancerous and precancerous lesions [8]. Dentists’ routine systematic examination of high-risk areas for malignancy might play an important role in the opportunistic screening of patients, particularly those who are in high-risk groups [8].

Long wait-times for treatment induces substantial anxiety and dissatisfaction in patients and supporting family members. Our study identified that patients waited an average of 13 weeks during the *pre-treatment interval* before starting treatment. According to the healthcare delivery practice guideline for head and neck cancer patients in Alberta, “… patients should be seen by a defined experienced surgeon with access to the necessary diagnostic tools within 2 weeks of referral… [and] patients undergoing primary surgical therapy should have surgery performed within 4 weeks of the ready-to-treat date” [46]. Although our findings were calculated from the date of diagnosis rather than from the referral date (which was missing for almost 35% of cases [12 out of 34]), the 13-week average is much longer than the acceptable timeline based on the Alberta guideline. A study conducted in Brazil showed the similar 12-week waiting time for initial treatment of patients diagnosed with head and neck cancers [47]. In addition, the lack of availability and overbooking of experienced surgeons identified in our study raises additional concerns for meeting the guideline’s recommendations.

Along with patient and professional factors, other factors also interfered with earlier diagnosis of oral and oropharyngeal cancers. This study has shown multiple behavioural, biological, and sociodemographic risk factors admitted by patients that highlight their vulnerability during clinical investigation **(Table 3).** Similarly, tumor size and invasive behaviour could result in negative outcomes even with early diagnosis and access to standard care, as has been seen in this study and is supported by the literature [8].

## Study limitation

There are some limitations inherent in retrospective studies using data from already recorded resources including potentially missing information [18]. However, clinical chart reviews have considerable advantages, in that they are less time-consuming and are a relatively inexpensive way to generate hypotheses that could be tested prospectively [48]. Patients’ consultation notes are unique resources to explore challenges faced by patients diagnosed in the late stages of disease, as many of these patients were deceased at the time of study. According to our preceding conducted study, Oral Cancer Surveillance and Control in Alberta: A Scoping Review [24], about half (47.9%) of total diagnosed OC and OPC patient cases were deceased at the time of our data collection.

In addition, the information provided through the Alberta Cancer Registry’s Initial Consultation Notes of the patients did not follow a consistent format across several different cases. Some of these cases were missing important data, such as the category of HCP at the first visit (physician, dentist, or specialist), and important dates, such as referral to the oncologist. The identified inconsistent ICNs were excluded to ensure the reliability of the study. Furthermore, this study is cautious to overinterpret the finding given relatively small dataset included in this study analysis.

## Conclusions

Our study showed an increase in time intervals for the five generated categories. The main contributors to diagnosis delay identified in this research were patients’ general lack of awareness regarding early symptoms of oral cancer and high-risk anatomic areas, inaccurate clinical judgement of attending physicians and dentists, and lengthy access to care. A sustainable plan is needed for both public awareness interventions and the implementation of a solid curriculum for the training of medical and dental students in order to enhance their knowledge, clinical judgement competency, and treatment management. Additionally, a mandatory integration of opportunistic screening of oral lesions as part of routine practice. This study has shown a lack of format and data consistency through the Initial Consultation Notes retrieved from the cancer registry database, which needs to be considered in future similar studies.
